# [Retracted] MicroRNA-363-3p is downregulated in hepatocellular carcinoma and inhibits tumorigenesis by directly targeting specificity protein 1

**DOI:** 10.3892/mmr.2025.13699

**Published:** 2025-09-29

**Authors:** Jie Ying, Xuechun Yu, Chaojian Ma, Yongqi Zhang, Jingwu Dong

Mol Med Rep 16: 1603–1611, 2017; DOI: 10.3892/mmr.2017.6759

Following the publication of this paper, it was drawn to the Editor's attention by a reader that, regarding the Transwell assay data shown in Fig 5, there was a slight concern that the data panels showing the results for the migration and invasion assay experiments for the two investigated cell lines (Hep3B and SMMC-7721) may have respectively originated from the same photos. Moreover, the ‘Hep3B/Migration/miR-363-3p mimics + pcDNA3.1-Sp1’ and SMMC-67721/Invasion/miR-363-3p mimics + pcDNA3.1-Sp1’ data panels contained an area of overlapping data, such that data which were intended to show the results from differently performed experiments had apparently been derived from the same original source. A subsequent analysis of the data in this paper revealed that there were a number of internal data duplications comparing Figs. 2C and 5 in this paper, and certain of these data had already been submitted for publication in an article written by different authors at different research institutes to the journal *Oncology Letters*.

In view of the fact that the abovementioned data had already been submitted for publication before this paper was submitted to *Molecular Medicine Reports*, the Editor has decided that this paper should be retracted from the Journal. The authors were asked for an explanation to account for these concerns, but the Editorial Office did not receive a reply.. The Editor apologizes to the readership for any inconvenience caused.

